# IMmuneCite: an integrated workflow for analysis of immune enriched spatial proteomic data

**DOI:** 10.21203/rs.3.rs-4571625/v2

**Published:** 2024-07-09

**Authors:** Arianna Barbetta, Sarah Bangerth, Jason T.C. Lee, Brittany Rocque, Evanthia T Roussos Torres, Rohit Kohli, Omid Akbari, Juliet Emamaullee

**Affiliations:** University of Southern California; University of Southern California; University of Southern California; University of Rochester; University of Southern California; Children’s Hospital of Los Angeles; University of Southern California; University of Southern California

**Keywords:** Single Cell Proteomics, Spatial Biology, Informatics Pipeline, Immune Microenvironment

## Abstract

Spatial proteomics enable detailed analysis of tissue at single cell resolution. However, creating reliable segmentation masks and assigning accurate cell phenotypes to discrete cellular phenotypes can be challenging. We introduce IMmuneCite, a computational framework for comprehensive image pre-processing and single-cell dataset creation, focused on defining complex immune landscapes when using spatial proteomics platforms. We demonstrate that IMmuneCite facilitates the identification of 32 discrete immune cell phenotypes using data from human liver samples while substantially reducing nonbiological cell clusters arising from co-localization of markers for different cell lineages. We established its versatility and ability to accommodate any antibody panel and different species by applying IMmuneCite to data from murine liver tissue. This approach enabled deep characterization of different functional states in each immune compartment, uncovering key features of the immune microenvironment in clinical liver transplantation and murine hepatocellular carcinoma. In conclusion, we demonstrated that IMmuneCite is a user-friendly, integrated computational platform that facilitates investigation of the immune microenvironment across species, while ensuring the creation of an immune focused, spatially resolved single-cell proteomic dataset to provide high fidelity, biologically relevant analyses.

## Introduction

High-throughput spatial imaging technologies, including Imaging Mass Cytometry (IMC) and Multiplexed Ion Beam Imaging Technology (MIBI), have allowed quantification of protein expression at single-cell resolution alongside robust analysis of spatial interactions due to the preservation of native tissue architecture. Indeed, these platforms have been used to characterize immune microenvironments associated with tumor biology, infectious processes, and inflammatory diseases through simultaneous detection of more than 40 protein antigens^1–8^. Data generated by this technology consist of a set of images, one for each measured metal ion channel, which are then analyzed using different computational biology algorithms^9^. Although spatial proteomics represent a powerful technology with growing use in biomarker discovery and therapeutic monitoring, its widespread adoption has been hampered by two major challenges: the presence of image artifacts, which can deteriorate the quality of data, and the choice of computational approach to perform reliable cell segmentation and assign cell phenotypes^2,10–18^. This is particularly relevant when examining immune microenvironments within tissue sections, where many different cell types, each with multiple phenotypic markers, coexist within an inflammatory lesion.

Similar to traditional immunohistochemistry (IHC), spatial proteomics can be performed on small and archival tissue samples including formalin-fixed paraffin-embedded (FFPE) or frozen tissues, and requires careful preparation of tissue sections and meticulous tissue staining, including antibody validation and titration to avoid image artifacts^9,19–21^. Other sources of artifacts, which can impair data quality and impede downstream analysis, are specific to spatial proteomics. These are classified into three types, including: channel spillover (or channel crosstalk), hot pixels, and shot noise (or noise). Channel crosstalk is due to metal isotopic impurity, oxide formation, and abundance sensitivity^22–24^. Sources of ‘noise’ can be related to non-specific antibody binding, ion counting imaging-based technology, antibody concentration, and tissue quality. Lastly, ‘hot pixels’ derive from the deposition of antibody aggregates on the tissue that are not associated to biological structures but cause areas with high ion counts, leading to erroneous signal interpretation. Thus, overcoming these image artifacts remains an important step in data pre-processing to obtain biologically valid conclusions.

Several methods have been developed to address image artifacts and pre-processing in spatial proteomics experiments^25^. Some allow for spillover correction only, as in the case with the R-based package CATALYST^23,26^. A semi-automated Ilastik-based method, and more recently, the IMC-Denoise pipeline based on the self-supervised deep learning-based shot noise image filtering (DeepSNiF) algorithm, were both developed to correct for technical and sample-specific noise^27,28^. IMC-Denoise also allows for hot pixel removal by applying differential intensity map-based restoration (DIMR)^28^. Conversely, in most cases, correction of hot pixels has been performed using thresholding methods^26,29,30^. More recently, SPEX (Spatial Expression Explorer), a modular and customizable pipeline, allows for channel spillover correction and denoising by applying global background correction, median filter denoising, and non-local means (NLM) denoising^31^. Currently, only MAUI (MBI Analysis User Interface), a MATLAB based user-friendly interface pipeline enables correction of all three types of image artifacts, channel crosstalk, noise, and hot pixels^32^. Together, these analytic tools can overcome challenges related to image pre-processing in spatial proteomics. However, data formatting challenges across multiple platforms, some of which are not free and open-source (e.g. MATLAB), advanced bioinformatics expertise across each of these platforms, and the need for deep knowledge of normal and abnormal tissue architecture, pathophysiology, and immunology, make these software cumbersome to apply to studies examining the immune microenvironment ^32^.

After pre-processing IMC data, the assignment of cell phenotypes (identification and classification) remains one of the most challenging tasks in spatial proteomics, particularly when studying the immune microenvironment. This is due to close proximity of cells which can cause lateral spillover of the signal from one object into another, especially in areas with dense immune infiltrates, where the cell-to-cell interaction creates physical overlap of cell membranes and cytoplasm, or where the overlapping of cell fragments can create a mismatch of nuclear signals and membranes^13,16^. Additionally, irregular cell shapes (e.g., macrophages, dendritic cells) represent another cause of lateral signal spillover from one cell mask into an adjacent cell mask. This can result in non-biological co-expression patterns (e.g., CD4/CD68, CD3/CD20, CD66/CD4) which lead to the identification of implausible immune cell phenotypes. Correction of lateral spillover was attempted with the development of RedDSEA, a MATLAB-based algorithm^33^. However, it has a limited ability to correct for lateral spillover in the case of multiple overlapping cells, is unable to perform cell clustering, and its performance depends on quality of image segmentation^33^.

Finally, cell phenotyping of pre-processed single cell datasets generated from spatial proteomics relies on either unsupervised or supervised clustering methods^34–39^. Unsupervised clustering approaches (e.g. FlowSOM, Phenograph, Gaussian mixture model) require manual annotation of each identified cluster, which comes with the arbitrary assignment of phenotype, including those with confounding marker expression patterns^34–36,40^. Supervised algorithms require a *priori* knowledge of marker expressions or the creation of a ground-truth dataset, thus limiting both the identification of novel or rare cell phenotypes and an in-depth characterization of cell status^37–39,41^. Additionally, these algorithms have been developed for cell suspension assays such as single-cell RNA sequencing (scRNA-seq), flow cytometry, and Cytometry by Time-Of-Flight (CyTOF), which lack the cell-to-cell spatial interaction component and are not affected by lateral spillover^34–41^. Overall, few algorithms have been specifically designed for cell classification in spatial proteomics assays, with none being immune-focused^42–46^.

To overcome these challenges, we designed IMmuneCite, a workflow that encompasses imaging pre-processing and a semi-supervised clustering algorithm for optimization and analysis of immune-enriched single-cell proteomic data generated via multiplexed imaging technologies. This is achieved through a newly developed Python pipeline, which includes conversion of single channel information contained in raw mcd files into tiff files ready for image artifact removal, performed in a streamlined, three-step approach based on previously described and newly implemented algorithms. Cell phenotyping occurs in two steps: the first relies on a supervised algorithm to identify metaclusters (general immune phenotypes such as CD4-T-cells, macrophages, neutrophils as well as non-immune phenotypes) while a second unsupervised algorithm enables the identification of specific subclusters and a more in-depth cellular status characterization. We demonstrate that image pre-processing facilitates downstream cell classification and identification of different cell phenotypes, while our clustering pipeline offers a robust and detailed description of the wide spectrum of immune cell phenotypes associated with each tissue pathology in clinically relevant human liver tissue. To demonstrate the robustness of the IMmuneCite pipeline across different diseases, antibody panels, and species, we externally validated its performance using publicly available IMC data generated in a mouse hepatocellular carcinoma (HCC) model. Ultimately, IMmuneCite offers a user-friendly, open-source computational resource with both human and murine-specific analysis pipelines to facilitate high quality, biologically accurate, and streamlined analysis of immune-focused spatial proteomics data.

## Results

### Overview of IMmuneCite workflow

**Figure 1:**
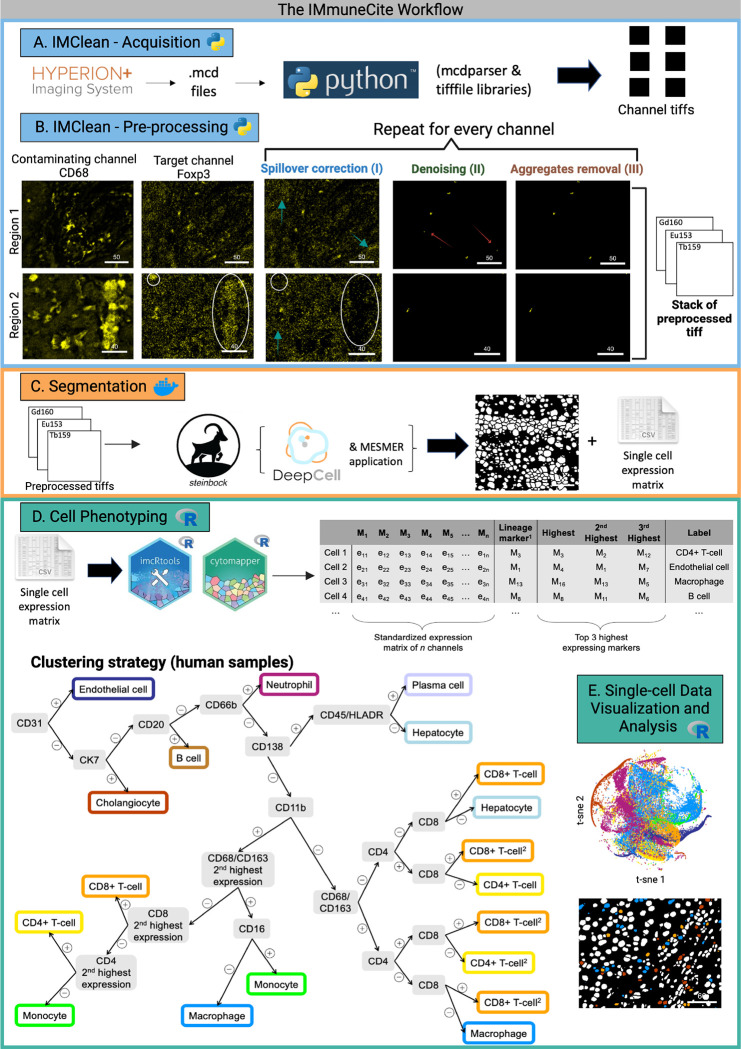
Overview of the IMmuneCite workflow for spatial proteomic data consisting of pre-processing (IMClean, blue), segmentation (orange), and cell phenotyping (green). **A.** Raw data (IMC mcd files) are imported into the IMClean pipeline and converted into .tiff files (a single .tiff file corresponds to a single channel). **B.** Each single channel image is processed in a three-step approach: channel spillover correction (or channel crosstalk removal), denoising, and aggregate removal. For example: in region 2, the raw image shows two areas of channel spillover (white ovals), which are corrected for in the first processing step (background removal). The green arrows point at areas of unspecific signal (noise) corrected for in the second imaging processing step (denoising). Red arrows (region 1 image) highlight antibody aggregates that are removed during the final step (aggregates removal). Afterwards, a stack of tiffs is created for each tissue section (also known as ROI) to include each channel to be used for analysis and is ready for image segmentation. **C.** IMClean-processed images are segmented using Mesmer to obtain single-cell masks and expression matrix to use for downstream analysis. **D.** Marker expression measurements are read into R and used for cell phenotype assignment using our IMmuneCite clustering algorithm for human samples. Information on the top three highest expressed markers is extracted and used for cell categorization and metaclusters phenotype assignment based on the algorithmic tree schematized in D. (1Needs to have a positive value; ^2^To account for imperfect CD4 staining (cells co-expressing CD4 and CD8) and spillover of signal from macrophages (due to their shape) into adjacent cell masks (cells co-expressing CD68/CD163 and CD4/CD8) E. Finally, single-cell data can be statistically compared, and cell phenotype can be visualized onto the mask of the corresponding tissue section (Scale bar unit = mm).

IMmuneCite represents a three-step framework that allows pre-processing of raw image files and cell identification through the integration of previously established and newly developed tools to create an accurate single-cell proteomic dataset to feed into downstream statistical and spatial analyses (Fig. 1). Each step – pre-processing, segmentation, and cell phenotyping – has been implemented as a standalone tool running on freely available platforms: Python, Docker, and R.

The first step of the IMmuneCite pipeline, *IMClean*, consists of image pre-processing and takes place in Python (Fig. 1, **blue**). Image pre-processing starts with data acquisition by transforming mcd files, which contain multi-channel images, into single channel tiff file images (Fig. 1A). The result is one folder for each sample or region of interest (ROI) containing as many tiff files as antibodies used for staining. In the second stage, data extracted in the form of single channel images are processed following a three-step approach: channel spillover correction, denoising, and aggregate removal (Fig. 1B). The first step allows for correction of channel(s) crosstalk. Signal from marker(s) spilling into other channels is capped, smoothed, and binarized to create a binary mask. This mask is then used to subtract a fixed value from all the pixels in the target channel that were positive in the contaminant channel^32^. The second step allows for a general noise removal from each channel by applying the minimum filter to cap the signal followed by a smoothing filter to detect and zero out noise. Lastly, aggregates – pixel spots of small size with high ion counts – are corrected by using a combination of image blurring using a Gaussian filter and size thresholding^32^. Once the signal from each marker has been properly cleaned, single channel images can be combined to create a single image stack.

In the second step of the IMmuneCite workflow, cell segmentation is performed on the single image stacks using the customizable Steinbock toolkit in Docker. Here, the user can alternatively select between two cell segmentation approaches: Ilastik/Cellprofiler, a supervised pixel classification method, or Mesmer, a completely automated and pre-trained deep-learning-enabled segmentation algorithm^47–50^ (Fig. 1C). In the current study, cell segmentation was performed using Mesmer. Segmentation outputs consist of single-cell data (including expression matrix as well as morphological and spatial features), segmentation masks, and antibody signal images ready to be used for cell phenotyping and downstream analysis performed in R.

The third and final step of the IMmuneCite pipeline enables cell phenotype identification (Fig. 1, **green**). This step consists of a semi-supervised approach with a two-part structure. The first part identifies metaclusters, which represent general cell phenotypes and are recognized using lineage markers (e.g., CD4, CD8, CD68, CD163, CD20). For each cell, information on lineage marker and the top three expressed markers are extracted and used to assign cell phenotype by using a combination of user-defined thresholds for the lineage markers of interest and logical operators (Fig. 1D). The accuracy of the threshold is confirmed by examining the overlapping of the channel signal and the identified cell cluster projected onto tissue masks. In this experiment, hepatocytes were identified via negative selection due to the lack of a specific marker in the panel. Thus, cells were assigned to the hepatocytes compartment if they had a low expression of most of the markers or high levels of CD138 only, as Syndecan-1 (CD138) is normally expressed in hepatocytes^51^. After careful examination, we also assigned any cell not falling under any of the defined metaclusters to the hepatocyte compartment. The second part identifies cell subclusters by using the unsupervised FlowSOM-based algorithm and the expression level of user-defined functional markers, thus providing in-depth information about the multiplexed status of cells within the same metacluster. Compared to fully unsupervised algorithms, this method allows for a reduction of cells with simultaneous expression of different lineage markers which would result in false annotation or implausible immune cell phenotypes (**Supplementary Fig. S1A and S1B**). Additionally, this detailed phenotyping has been shown to facilitate the identification of rare cell populations unique to certain disease states which would otherwise remain unnoticed when using solely unsupervised clustering algorithms **(Supplementary Fig. S1C**)^52^. Lastly, distinct cell types are used for downstream statistical comparison across different experimental conditions and more advanced spatial analysis (Fig. 1E)^46,48^.

#### Quantification of clustering recognition improvement after IMmuneCite application

**Figure 2:**
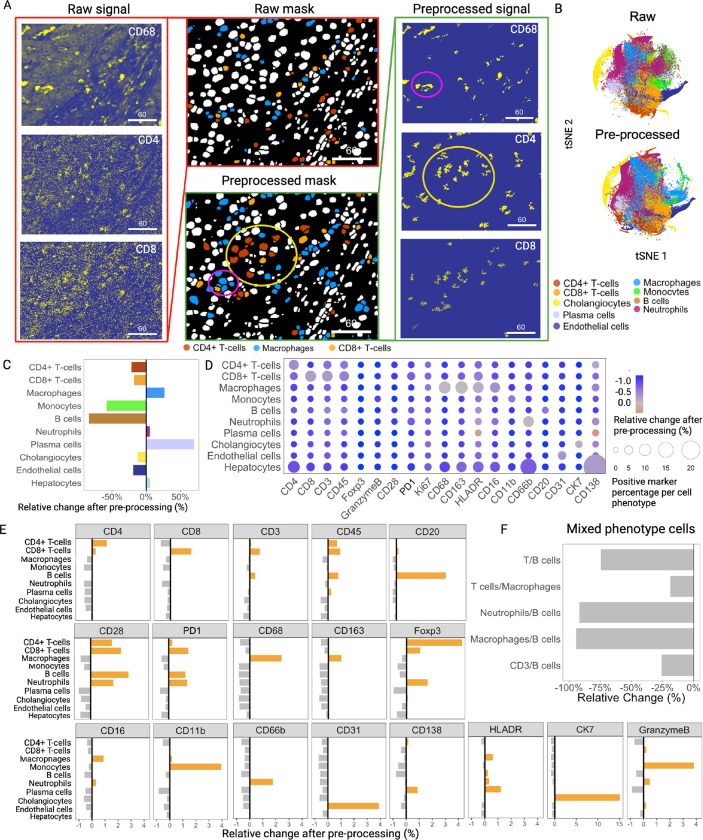
The IMmuneCite workflow facilitates and improves phenotyping of immune cells within immune enriched human liver tissue. **A.** Example of representative CR liver tissue section showing raw signal and IMClean-processed signal; IMClean enhances the identification of CD4^+^ T-cells, CD8^+^ T-cells, and macrophages compared to the same raw signal image as shown in the corresponding cell masks (Scale bar unit = mm). **B**. t-SNE plots showing differences in metacluster density and distribution in raw vs IMClean-processed TCMR data. **C**. Relative change in cell percentage within each metacluster before and after image pre-processing; IMClean increased the number of macrophages, plasma cells, neutrophils, and hepatocytes identified in the human liver rejection IMC dataset. **D.** IMClean reduces nonspecific marker signal while enhancing the specific ones within the appropriate cell types. The circle size indicates the positive marker percentage in a particular phenotype, and the circle color indicates the relative change of the positive rate for a particular marker after pre-processing. **E**. IMClean pre-processing increases the specificity of the immune metacluster phenotyping; in relation to each marker, the ratios of specific metaclusters expressing a certain marker increase while the ratios of non-specific phenotypes for a particular marker decrease, thus showing a biologically appropriate correlation between markers and assigned metacluster. The relative change is defined as the difference in percentage composition of each cell type between IMClean-processed and raw data. F. IMClean reduces the frequency of cells showing mixed phenotypes – cells that express markers belonging to different cell lineages (e.g. CD20 and CD8, or CD68 and CD4) – thus decreasing the rate of non-biological immune phenotypes.

To evaluate whether the application of IMmuneCite improves image quality and facilitates cluster identification, we applied our workflow to a biobank of human liver rejection samples^52^. This dataset included 96 IMC images consisting of 24 no rejection (NR) liver core biopsies, 41 needle core biopsies with proven acute T-cell mediated rejection (TCMR), and 14 chronic rejection (CR) samples. FFPE tissue samples were stained using a customized 22-antibody panel (**Supplementary Table S1**). We used these multiplexed images to generate two distinct single-cell datasets for comparison. The first dataset (461,816 cells) was obtained after pre-processing all IMC images using IMClean, the first step in the IMmuneCite workflow. We performed channel crosstalk correction, utilizing CD68 as the contaminating marker, followed by denoising and aggregate removal for each of the markers in the panel (Fig. 1A–B). We optimized image artifacts correction for each channel (**Supplementary Fig. S2**). Thus, true marker signal was enhanced but not removed (**Supplementary Fig. S2A**). For example, both raw images and images where artifacts were not properly corrected, still presented CD68 and FoxP3 signal overlap, which would result in the presence of FoxP3 + macrophages (**Supplementary Fig. S2B-D**). Conversely, an aggressive correction would result in removal of true signal, affecting overall macrophage identification (**Supplementary Fig. S2E**). The second dataset was obtained by segmenting the same 96 raw images with no correction of artifacts and contained a total of 402,287 cells. Correlation analysis showed that a similar number of cells was obtained after segmentation of raw vs. pre-processed IMC data (Spearman correlation = 0.97). Subsequently, our cell phenotype identification algorithm was applied to both datasets to identify metaclusters and subclusters (Fig. 2). The thresholds for the lineage markers were optimized for each dataset separately to guarantee the most reliable cell phenotyping in each condition. By visually inspecting the signal of multiple markers in raw vs. pre-processed images and the corresponding clusters on image masks, we observed improvement of image quality, which enhanced metacluster identification (Fig. 2A). Additionally, an improved overlap between lineage marker signal and the corresponding cell plotted on the segmented tissue mask was observed in IMClean-processed images (Fig. 2A). Differences between the two datasets in cell distribution and density for each assigned phenotype was also visible when data were plotted in two dimensions using t-Distribution Stochastic Neighbor Embedding (t-SNE) (Fig. 2B, **Supplementary Fig. S1D**). After IMClean pre-processing, we observed a decrease in the following metaclusters: CD4 + T-cells (22%), CD8 + T-cells (18%), B cells (84%), monocytes (58%), cholangiocytes (12%), and endothelial cells (19%). Conversely, macrophages, plasma cells, and neutrophils increased by 26%, 70%, and 5%, respectively (Fig. 2C).

We then investigated how IMClean changes the marker expression pattern within each metacluster. In the pre-processed dataset, we observed a positive percentage of cells expressing phenotype-specific markers in each metacluster, while the percentage of markers not related to the phenotype was minimal (Fig. 2D, **circle size**). For example, cells in the CD4 + T-cell population positively expressed both CD4 and CD3. Similarly, cells in the CD8 + T-cell metacluster were positively expressing CD8 and CD3 and cells in the macrophage metacluster were positively expressing CD68, CD163, HLADR (Human leukocyte antigen-DR), and CD16. On the other hand, the proportion of cells expressing markers not specific to these metaclusters such as CD20, CD31, and CK7 was near zero. When compared to the raw dataset, we observed an overall decrease in relative change after pre-processing in the proportion of cells expressing unspecific markers for each metacluster (Fig. 2D, **color scale**). This suggests that IMClean increased the sensitivity of immune cell phenotyping.

Additionally, after image pre-processing, the expression of each marker was enriched in the specific cell phenotype while decreased in other non-specific phenotypes, suggesting an increased specificity of the phenotypic marker for each metacluster (Fig. 2E, **Supplementary Table S2**). For instance, post-IMClean, we observed an increased ratio of CD20-expressing cells in the B cell metacluster (relative change = + 11.31%), while the proportion of CD20-expressing cells decreased in the other metaclusters. Similarly, CD66b-expressing cells were enriched in neutrophils (+ 1.77%), while the proportion of CD66b-expressing cells was reduced in other metaclusters. CD68, CD163, and CD16-expressing cells all increased in the macrophage metacluster (+ 2.41%, + 0.98%, and + 0.9%, respectively), while the proportion of cells expressing these markers largely decreased in the other metaclusters. The ratio of CD11b-expressing cells increased in the monocyte metacluster (+ 3.94%), while it decreased in other metaclusters. We also observed a greater proportion of CD4 and CD8-expressing cells in the corresponding metaclusters (+ 1.12% and + 1.60%, respectively), while their expression decreased in other cell phenotypes. However, we noticed a small percentage of CD3-expressing cells in the B cell metacluster (+ 0.40%) and a small percentage of CD8-expressing cells in CD4 + T-cell compartment (+ 0.26%) likely due to the close proximity of those cells in immune enriched tissue as is the case of TCMR post-liver transplant, which causes lateral spillover of the signal from one cell mask into the adjacent cell mask^33^. We also observed increased ratios of CK7-expressing cells in the cholangiocyte metacluster (+ 15.12%) and CD31-expressing cells in the endothelial cell compartment (+ 3.9%). Lastly, for markers not restricted to a single cell lineage, we observed an overall positive relative change in biologically appropriate metaclusters, with few exceptions such as CD28, perhaps due to the poor staining observed for this antibody (Fig. 2E).

We also evaluated whether IMClean reduced the frequency of cells showing mixed phenotypes, defined as cells expressing high levels of two different lineage markers, impacting assignment of cell phenotype and potential false annotation. IMClean reduced the frequency of mixed phenotypes, with a reduction of 74.3% in the co-expression of B and T cell markers (CD4 or CD8). Similarly, the co-expression of CD3 and CD20 had a 25.5% reduction after image pre-processing (Fig. 2F).

#### IMmuneCite facilitates the identification of T-cell, B cell, and Monocyte-Macrophage subclusters offering detailed description of cell states in human liver rejection samples

**Figure 3:**
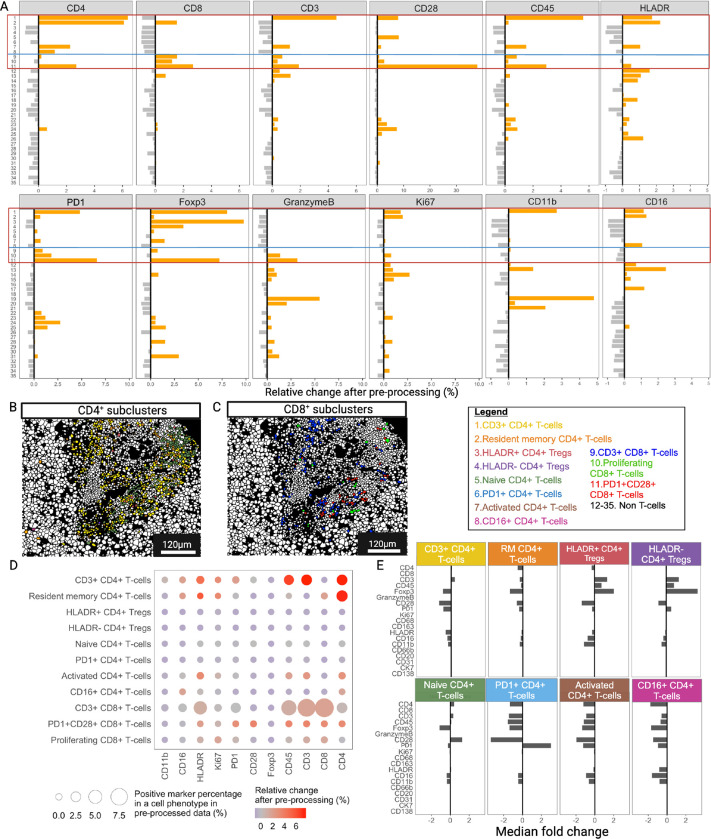
The IMmuneCite workflow enhances T lymphocyte subcluster identification and provides details on cell activation states. **A.** IMClean pre-processing increases the specificity of the immune subcluster phenotyping; for each marker, the ratio of each specific subcluster increases while those of non-specific phenotypes decrease, making cell phenotype and expressed markers biologically appropriate. For example: after IMClean pre-processing, ratio of cells with a positive PD1 expression was increased in CD4^+^ and CD8^+^ T-cell subclusters while a negative (decrease) ratio of PD1 positive cells was observed in non-T and B-cell subclusters (Subclusters 12 – 35 = 12: M1 macrophages; 13: M2 macrophages; 14: Proliferating M1 macrophages, 15: Proliferating M2 macrophages; 16: CD16^+^ M1 macrophages; 17: CD16^+^ M2 macrophages; 18: HLADR^+^ M2 macrophages; 19: Classical monocytes; 20: Intermediate monocytes; 21: Activated monocytes; 22: B cells; 23: Proliferating B cells; 24: PD1^+^ B cells; 25: Neutrophils; 26: Plasma cells; 27: Cholangiocytes; 28: Proliferating Cholangiocytes; 29: HLADR^+^ Cholangiocytes; 30: Endothelial cells; 31: Proliferating Endothelial cells; 32: HLADR^+^ Endothelial cells, 33: Hepatocytes; 34: Proliferating Hepatocytes; 35: HLADR^+^ Hepatocytes). **B.** Representative zoomed-in liver tissue section highlighting CD4^+^ T-cells colored by cell subpopulation (see color key legend). Among the subpopulations identified via unsupervised clustering within the CD4^+^ T-cell metacluster in both the raw and preprocessed datasets, eight emerged to be common to both datasets: Resident Memory CD4^+^ T-cells, CD3^+^CD4^+^ T-cells, Activated (HLADR^hi^) CD4^+^ T-cells, CD16^+^CD4^+^ T-cells, Naïve CD4^+^ T-cells, HLADR^+^CD4^+^ Tregs, HLADR-CD4^+^ Tregs, and PD1^+^CD4^+^ T-cells. **C.** Representative zoomed-in liver tissue section highlighting the CD8^+^ compartment (see color key legend); after using unsupervised clustering algorithm, three CD8^+^ T-cell subclusters were identified to have the same expression patterns in both the raw and the IMClean-processed datasets (CD3^+^CD8^+^ T-cells, Proliferating (Ki67^+^) T-cells, and PD1^+^CD28^+^ T-cells) for which marker expressions were compared before and after IMClean pre-processing (as show in A). D. Comparison of marker expression between raw and IMClean-processed T-cell subclusters showed that IMClean reduces non-specific marker signal while enhancing the specific ones within cell types. The circle size indicates the positive marker percentage in a particular phenotype, and the circle color indicates the relative change of the positive rate for a particular marker after pre-processing. **E.** Median fold change of marker expression between raw and IMClean-processed for CD4^+^ T-cell subclusters.

Spatial proteomics and, in particular, IMC, have been primarily applied to study the complexity of the tumor microenvironment^2,3,16,53^. Thus, being able to dissect the multiplicity of all immune cell phenotypes remains the principal scope of this technology, especially when applied to inflammatory and immune-mediated diseases, where uncovering rare cell types might be crucial. To assess whether IMClean affects the identification of immune cell subpopulations, we applied our IMmuneCite subclustering algorithm to the CD4 + and CD8 + T-cell metaclusters and compared the results between raw and pre-processed data (Fig. 3, **Supplementary Fig. S3**). We identified eight different CD4 + T-cell subclusters in raw data and nine subclusters in pre-processed data, with eight showing the same marker expression profile (Fig. 3A, **Supplementary Fig. S3A**). The frequency of these CD4 + T-cell subpopulations in pre-processed vs. raw data was also calculated (**Supplementary Fig. S3B**). Similarly, we identified four CD8 + T-cell subclusters within the raw dataset and five different CD8 + T-cell subtypes in the IMClean-processed data (Fig. 3A, **Supplementary Fig. S3C-D**). When assessing the expression of both lineage and functional markers, these were enriched in the specific cell phenotype while decreased in other non-specific phenotypes, suggesting an increase in phenotype specificity (Fig. 3A). For example, the ratio of CD4 and Foxp3-expressing cells increased the most in CD4 + T-cell subclusters such as CD3 + CD4 + T-cells and CD4 + Tregs, the proportion of CD8-expressing cells increased most in CD8 + T-cell subclusters, CD3 and CD45-expressing cells increased the most in T-cell subclusters, and the ratio of PD1 (programmed death 1)-expressing cells increased mostly in PD1 + subclusters. Conversely, the ratio of CD4, CD8, CD3, and CD45-expressing cells mostly decreased in non-T-cells. Additionally, the increased ratio of CD11b-expressing cells in CD3 + CD4 + T-cells is in agreement with their recent recruitment and activation at the inflammatory site^54^. Tissue sections showing the spatial distribution of these subclusters are presented in Fig. 3B and 3C. After IMClean pre-processing, we observed an enrichment of specific markers in each subcluster, while the expression of markers not specific for the subclusters decreased (Fig. 3D). For example, activated CD4 + T-cells showed an increase in cells expressing HLADR, CD3, CD4, and CD45, while proliferating CD8 + T-cells observed an increase in cells expressing Ki67. We also evaluated the median fold change for all markers for each CD4 + T-cell (Fig. 3E) and CD8 + T-cell subcluster (**Supplementary Fig. S3E**), showing a greater median expression of lineage specific markers after IMClean pre-processing (Fig. 3E). For instance, after pre-processing, the median expression of PD1 and Foxp3 increased in PD1 + CD4 + T-cells and Tregs, respectively.

**Figure 4:**
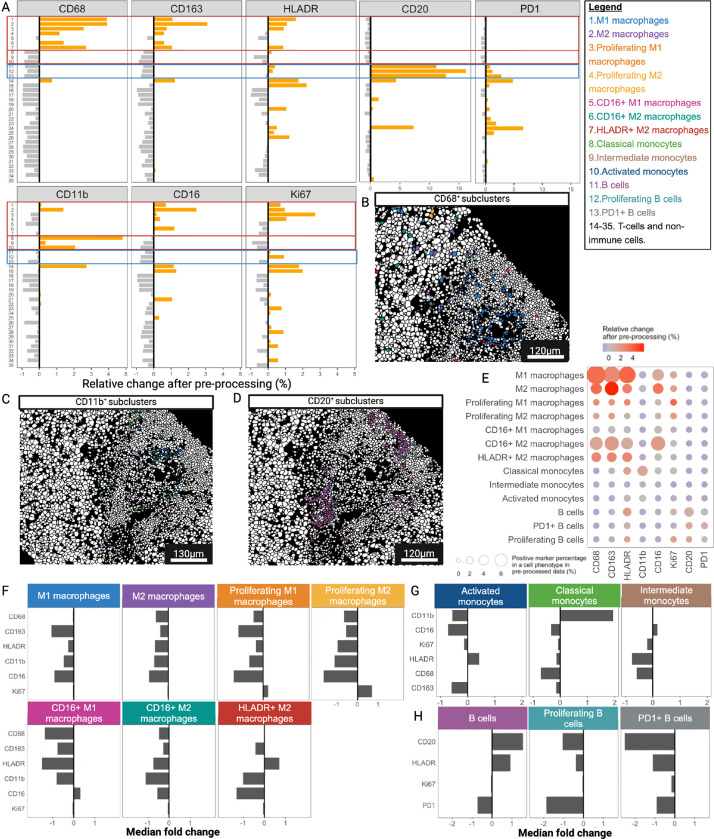
The IMmuneCite workflow enables an accurate phenotyping and depiction of cellular states of Monocyte, Macrophage, and B cell subclusters. **A.** IMClean pre-processing increases the specificity of subcluster phenotyping within the monocyte, macrophage, and B cell compartments; given a certain positive marker, the ratio of each specific subcluster (for that marker) increases while that of non-specific phenotypes decreases, making cell phenotype and expressed markers biologically appropriate. For example: after IMClean pre-processing, ratio of cells with a positive CD68 expression was increased only in macrophage subclusters while a negative (decrease) ratio of CD68 positive cells was observed in T and B-cell subclusters (Subclusters 14 – 35= 14: CD3^+^ CD4^+^ T-cells; 15: Resident memory CD4^+^ T-cells; 16: HLADR^+^ CD4^+^ Tregs; 17: HLADR-CD4^+^ Tregs; 18: Naïve CD4^+^ T-cells; 19: PD1^+^ CD4^+^ T-cells; 20: Activated CD4^+^ T-cells; 21: CD16^+^ CD4^+^ T-cells; 22: CD3^+^ CD8^+^ T-cells; 23: Proliferating CD8^+^ T-cells; 24: PD1^+^ CD28^+^ CD8^+^ T-cells; 25: Neutrophils; 26: Plasma cells; B cells; 23: Proliferating B cells; 24: PD1^+^ B cells; 25: Neutrophils; 26: Plasma cells; 27: Cholangiocytes; 28: Proliferating Cholangiocytes; 29: HLADR^+^ Cholangiocytes; 30: Endothelial cells; 31: Proliferating Endothelial cells; 32: HLADR^+^ Endothelial cells, 33: Hepatocytes; 34: Proliferating Hepatocytes; 35: HLADR^+^ Hepatocytes). **B.** Representative zoomed-in liver tissue section highlighting macrophages colored by cell subpopulation (see color key legend). Subpopulations were identified via unsupervised clustering within the macrophage metacluster in both raw and pre-processed datasets. Seven distinct subpopulations emerged to be common between the two datasets: M1 and M2 populations, Proliferating (Ki67^+^) M1 macrophages, Proliferating (Ki67^+^) M2 macrophages, CD16^+^ M1 macrophages, CD16^+^ M2 macrophages, and HLADR^+^ M2 macrophages. **C.** Representative zoomed-in liver tissue section highlighting monocyte subpopulations (see color key legend); after unsupervised clustering applied to both datasets, three subpopulations were identified to have the same expression patterns in both the raw and the IMClean-processed datasets: Classical monocytes (CD11b^+^), Intermediate (CD16^+^CD68^+^CD163^+^) monocytes and Activated (HLADR^high^) monocytes. **D.** Representative zoomed-in liver tissue section showing B-cell subclusters identified via unsupervised clustering in both raw and IMClean-processed datasets, which shared the following B-cell subpopulations: B cells (CD45^+^CD20^+^HLADR^+^), PD1^+^ B cells (CD45^+^CD20^+^HLADR^+^PD1^+^), and proliferating B cells (CD45^+^CD20^+^HLADR^+^Ki67^+^). **E.** Comparison of marker expressions between raw and IMClean-processed for monocyte, macrophage, and B cell subclusters showed that IMClean reduces non-specific marker signal while enhancing the specific ones within cell types. The circle size indicates the positive marker percentage in a particular phenotype, and the circle color indicates the relative change of the positive rate for a particular marker after pre-processing. **F-H.** Median fold change of marker expression between raw and IMClean-processed for macrophage, monocyte, and B cell subclusters, respectively

We also evaluated differences in the expression patterns of all markers between the raw and IMClean-processed datasets after applying our IMmuneCite subclustering algorithm to cells within the macrophage, monocyte, and B-cell compartments (Fig. 4, **Supplementary Fig. S4**). We identified seven different subtypes of macrophages in the raw dataset while nine macrophage subclusters were detected in the IMClean-processed dataset, with a different percentage distribution (**Supplementary Fig. S4A-B**). Four different monocyte subtypes were identified in both datasets, with a greater frequency of classical monocytes found in pre-processed data (**Supplementary Fig. S4C-D**). The same three B-cell subtypes were identified in both datasets (**Supplementary Fig. S4E-F**). Similar to what was observed in the T-cell compartments, we observe an increase in phenotype specificity (Fig. 4A). We noticed that the ratio of CD11b-expressing cells was mostly enriched in monocyte subclusters: +2.06% in activated monocytes, + 4.80% in classical monocytes, and + 0.35% in intermediate monocytes (Fig. 4A). The ratio of cells expressing PD1 increased in B cell subclusters while decreasing in all non-B and non-T-cell subclusters (Fig. 4A). The phenotypes commonly identified in raw and IMClean-processed datasets were mapped back onto their segmentation masks (Fig. 4B–D). Furthermore, we observed that, after IMClean pre-processing, each subcluster had an increased expression rate of cells expressing biologically relevant markers, while the non-specific markers were reduced (Fig. 4E, **color scale**). For example, we observed that proliferating macrophages and proliferating B-cells showed an increase in cells expressing Ki67, while non-specific markers such as PD1 and CD11b were reduced. We also evaluated the median fold change of marker expression after pre-processing for macrophages, monocytes, and B-cells (Fig. 4F–H). Taken together, these results show the robustness of the IMmuneCite workflow to generate biologically accurate outputs when applied to human immunology experiments. IMmuneCite allowed the discrimination of cells in different states of activation in the CD4 + and CD8 + T-cell and the B cell compartments which suggest a complex immune response and cell-to-cell interaction within the alloimmune microenvironment during active TCMR episodes (Fig. 3–4, **Supplementary Fig. S3-S4**)^52^. Additionally, we were able to reveal differences in macrophage polarization and their polymorphic activation states (Fig. 4, **Supplementary Fig. S4**). Lastly, IMmuneCite allowed the detection of new molecular pathways important in mediating not only the alloimmune response, but also potentially new targets for immunotherapy to treat allograft rejection^52^.

### External validation of the IMmuneCite workflow and development of a murine IMC analysis pipeline

**Figure 5:**
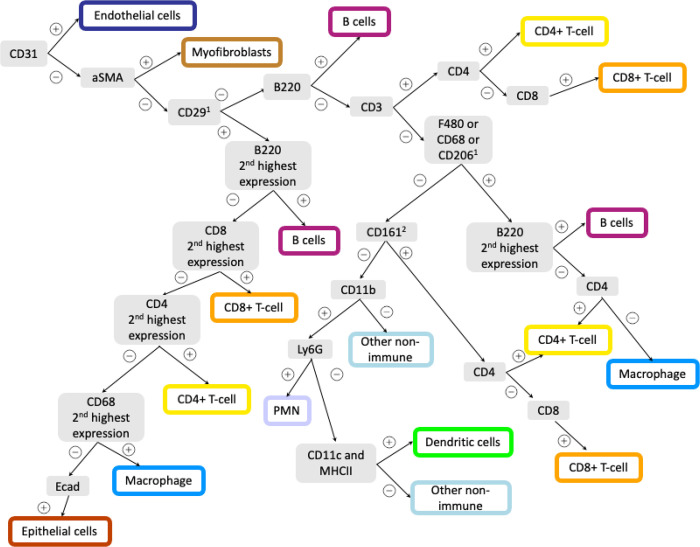
IMmuneCite clustering algorithm used to analyze IMC data obtained from HCC mouse model. The IMmuneCite clustering pipeline is robust across multiple disease’ immune microenvironments and species. We adapted our IMmuneCite clustering algorithm to analyze IMC data obtained from four different HCC mouse models. After IMClean pre-processing and segmentation of images, marker expression measurements contained in the single-cell expression matrix are read into R and used for cell phenotype assignment using the IMmuneCite clustering algorithm adapted for mouse samples. Information on the top three highest expressed markers is extracted and used for cell categorization and phenotype assignment based on the algorithmic tree schematized above. The top five highest expressed markers were used for macrophages identification. ^1^To account for broad and unspecific expression of CD29 and spillover of signal from macrophages (due to their shape) into adjacent cell masks (cells co-expressing CD11c or CD68/CD4 and CD68/B220).

**Figure 6:**
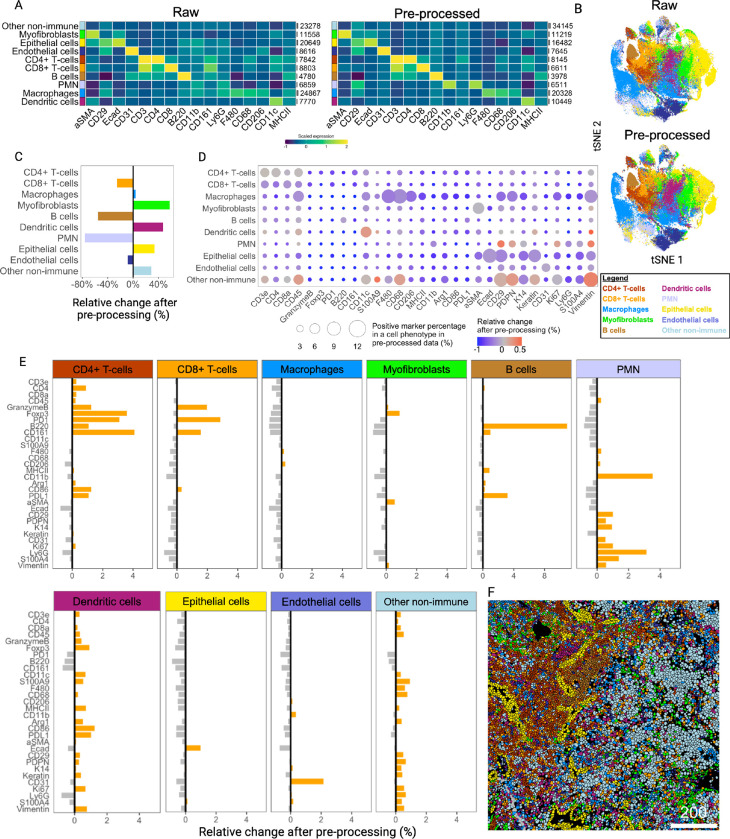
Validation of the IMmuneCite workflow in IMC data from murine HCC tissue demonstrates an enhancement in the quality of data in structurally complex immune enriched tissues. **A.** Heatmaps showing scaled marker expression within the 10 metaclusters identified in both raw and IMClean-processed external IMC mouse data, with grey bars indicating the total number of cells per cell type. **B.** t-SNE plots comparing raw and pre-processed data showing different density and distribution of cell metaclusters in raw vs IMClean-processed data. **C.** Relative change in cell percentage within each metacluster before and after image pre-processing; IMClean increased the number of macrophages, myofibroblasts, dendritic cells, and epithelial cells identified after image pre-processing. **D.** Within each metacluster, IMClean reduces non-specific marker signal while enhancing the specific ones for a particular cell phenotype. The circle size indicates the positive marker percentage in a particular phenotype, and the circle color indicates the relative change of the positive rate for a particular marker after pre-processing. **E.** IMClean pre-processing increases the specificity of the metacluster phenotyping; in relation to each marker, the ratios of specific metaclusters expressing a certain marker increase while the ratios of non-specific phenotypes for a particular marker decrease, thus showing a biologically appropriate correlation between markers and assigned metacluster. Relative percentage change was computed as positive cell (%) in the IMClean-processed data minus positive cell (%) in raw data divided by the total number of cells in the raw data. **F.** Representative tissue section showing the spatial location of the ten identified metaclusters, which highlights structural components and infiltrating immune cells within mouse HCC tissue (Scale bar unit = mm).

To assess its performance and versatility, we applied the IMmuneCite workflow to an external and publicly available IMC dataset containing 12 multiplexed images of liver tissues obtained from syngeneic mouse HCC models^14^. The FFPE slides were stained with a 35-antibody panel. We generated a raw dataset containing 125,222 cells along with a curated IMClean-processed dataset containing 125,790 cells. In both instances, cell segmentation was performed using Mesmer. We customized the IMmuneCite clustering algorithm to include the greater number of markers used in this study and maximize cell phenotyping. Metaclusters were identified using the mouse IMmuneCite clustering algorithm tree shown in Fig. 5, which led to the identification of 10 metaclusters, including 7 immune and 3 non-immune metaclusters (Fig. 6A). The thresholds for the lineage markers were optimized for each dataset separately to guarantee the most reliable cell phenotyping in each condition. Labelling accuracy was verified by visually inspecting the signal of multiple markers in raw and pre-processed images and the corresponding clusters on image masks. The differences in the expression profiles of these metaclusters are shown in the heatmaps (Fig. 6A), while differences in density and distribution between the two datasets are visualized using t-SNE plots (Fig. 6B). After applying IMClean pre-processing to the mouse data, we observed a decrease in the following metaclusters: CD8 + T-cells, B cells, Polymorphonuclear cells (PMNs), and endothelial cells. Conversely, macrophages, myofibroblasts, dendritic cells, epithelial cells, and other non-immune cells increased (Fig. 6C).

When we looked at the frequency of cells expressing markers biologically appropriate for the cell lineage in IMClean-processed data, we observed that cells in the CD4 + T-cell metacluster positively expressed both CD4 and CD3 and cells in the macrophage metacluster positively expressed CD68, F480, and CD206, while the proportion of cells expressing other non-specific markers in these metaclusters was minimal or null (Fig. 6D, **circle size**). Similarly, cells in the myofibroblast and dendritic cell metaclusters positively expressed αSMA and CD11c, respectively. Additionally, when we compared the expression of these cells in raw vs. IMClean-processed datasets, we saw that, within each metacluster, the expression of non-specific markers is mainly reduced, while the expression of specific markers is enriched, especially in dendritic cells and other non-immune cells (Fig. 6D, **color scale**). Moreover, the expression of both lineage and functional markers was enriched in the specific cell phenotype while decreased in other non-specific phenotypes, suggesting an increase in phenotype specificity (Fig. 6E). For example, post-IMClean, we observed an increase in the ratio of PD1-expressing cells in T-cells and a decrease in all other metaclusters. The ratio of cells expressing αSMA increased in myofibroblasts and epithelial cells, while decreasing in other metaclusters. The proportion of cells expressing B220 increased in B-cells, while decreasing in other metaclusters. The increased ratio of cells expressing B220 and cells expressing CD8a in the CD4 + T-cell compartment or the increased ratio of cells expressing CD8a and cells expressing CD3 in the dendritic cell metacluster could be due to cell segmentation and the close proximity among these APC and effector cells. Some discrepancies such as the increased presence of cells expressing epithelial markers in immune metaclusters might be due to their widespread staining and broader expression of those markers compared to immune specific markers, which cause overlap between them (**Supplementary Fig. S5A**). Importantly, we detected image artifacts related to the quality of tissue sections which complicated the clustering step given that a cluster was identified where all markers had high expression patterns (**Supplementary Fig. S5A**). However, we were able to visualize a wrinkle in the tissue section, leading to this artifact, and ultimately exclude cells from that specific area from further analysis given their non-biological expression pattern (**Supplementary Fig. S5B-C**). We also evaluated the frequency of cells with mixed phenotypes in mouse data with and without pre-processing. Again, IMClean pre-processing reduced the frequency of non-biological mixed phenotypes in the case of co-expression of B and T cell markers (CD4 or CD8), co-expression of CD3 and B220, and co-expression of dendritic cells and T or B cell markers (**Supplementary Fig. S5D**). A segmented mask showing the spatial location of the metaclusters obtained from the IMClean-processed dataset highlights the structural elements and the immune cell infiltration in a mouse HCC section (Fig. 6F).

**Figure 7:**
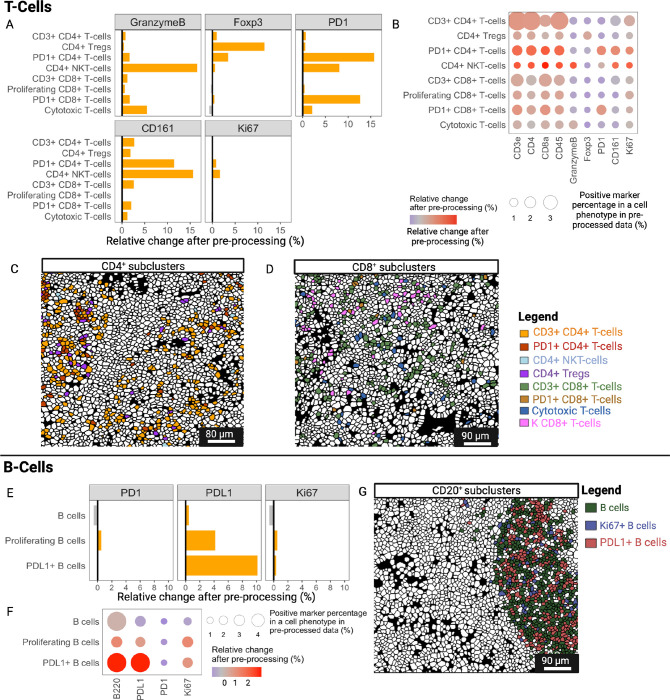
The IMmuneCite workflow allows discrete phenotyping of T and B-cell compartments in murine HCC tissue. **A.** Assessment of IMC data from HCC mouse models showed that the IMmuneCite workflow increases the specificity of CD4^+^ and CD8^+^ T-cell subcluster phenotyping; for each marker, the ratio of specific subclusters with positive expression increases while the ratio of non-marker specific phenotypes decreases. **B.** Comparison of marker expression between raw and IMClean-processed for CD4^+^ and CD8^+^ T-cells showed that pre-processing reduces the non-specific marker signal while the specific ones are enriched in their respective cell types. The circle size indicates the positive marker percentage in a particular phenotype, and the circle color indicates the relative change of the positive rate for a particular marker after pre-processing. **C.** Representative zoomed-in of mouse HCC liver tissue section highlighting CD4^+^ T-cells colored by cell subpopulation (see color key legend). Subpopulations identified via unsupervised clustering within the CD4^+^ T-cell metacluster in both the raw and processed datasets are CD3^+^ CD4^+^ T-cells, PD1^+^ (PD1^+^ CD3^+^) CD4^+^ T-cells, CD4^+^ (CD161^+^ Granzyme B^+^ CD3^+^) NKT cell, and CD4^+^ (CD3^+^ FoxP3^+^) Tregs. **D**. Representative zoomed-in mouse HCC liver tissue section highlighting CD8^+^ T-cell compartment (see color key legend); after using unsupervised clustering algorithm, four CD8^+^ T-cell subclusters were identified to have the same expression patterns in both the raw and the IMClean-processed datasets: CD3^+^CD8^+^ T-cells, Proliferating (Ki67^+^) T-cells, and PD1^+^ (CD3^+^) CD8^+^ T-cells, and Cytotoxic (Granzyme B^+^ CD3^+^ CD8^+^) T-cell. **E.** In the B cell compartment, IMClean increases the specificity of subcluster phenotyping; the ratio of specific subclusters with positive scaled expression for a certain marker increases while the ratio of non-specific subclusters decreases. **F.** Comparison of marker expressions between raw and IMClean-processed in B cell subclusters showed that IMClean reduces non-specific marker signal while enhancing the specific ones within cell types. The circle size indicates the positive marker percentage in a particular phenotype, and the circle color indicates the relative change of the positive rate for a particular marker after pre-processing. **G.** Representative case showing spatial distribution of B cell subclusters within mouse HCC tissue section including generic B cells (CD45^+^ B220^+^), Proliferating (Ki67^+^ CD45^+^) B cells, and PDL1^+^ (CD45^+^) B cells.

Following the same approach used for the human liver datasets, we performed subcluster phenotyping in both raw and pre-processed mouse datasets, obtaining 25 immune subclusters from raw data vs. 24 from IMClean pre-processed data (**Supplementary Fig. S5E-S6**). After IMClean pre-processing, the expression of functional markers biologically specific to CD4 + and CD8 + T-cell subclusters was enriched in these subclusters, suggesting an increase in phenotype specificity (Fig. 7A). For example, the proportion of Foxp3-expressing cells was enriched in the CD4 + Tregs subcluster and reduced in the other non-CD4 + T-cell subclusters. The ratio of PD1-expressing cells was greater in both CD4 + and CD8 + T-cells identified as PD1 + CD4 + T-cells and PD1 + CD8 + T-cells, respectively, and the proportion of GranzymeB-expressing cells was increased in Cytotoxic T-cells and CD4 + natural killer T-cells (NKT-cells). Additionally, after IMClean pre-processing, we noticed an enrichment of specific markers for each subcluster, while the expression of markers not specific for the subclusters decreased (Fig. 7B). Tissue sections representing CD4 + and CD8 + T-cell subclusters are shown in Figs. 7C and 7D. While the same three B-cell subclusters were identified in both datasets, we found that the expression of functional markers was enriched in these biologically specific subclusters (Fig. 7E). The frequency of cells expressing subcluster specific markers increased in the cell type resulting in increased relative change, while the non-specific markers decreased (Fig. 7F, with visualization in Fig. 7G).

**Figure 8:**
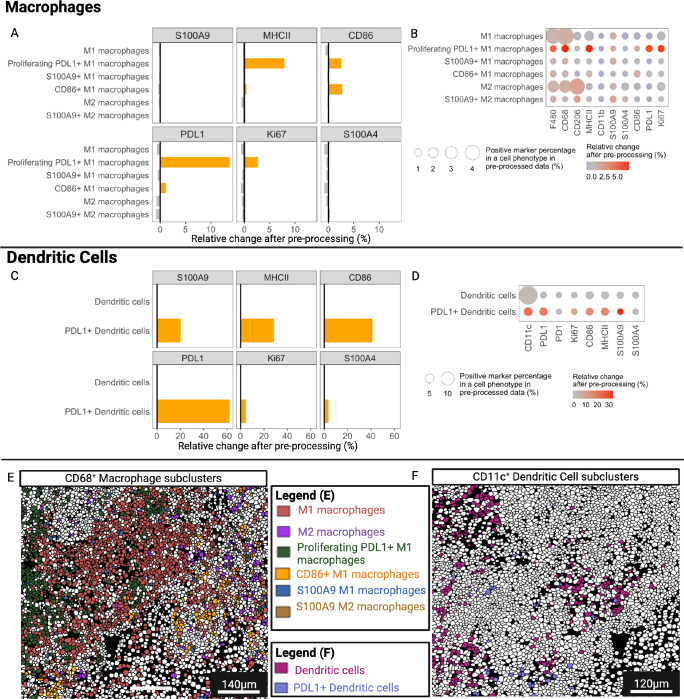
The IMmuneCite workflow enables an accurate phenotyping of macrophages and dendritic cells in mouse HCC tissue. **A-B.** The IMmuneCite workflow ameliorates both specificity (A) and sensitivity (B) of macrophage sub-phenotyping; for a certain marker with a positive scaled expression, the ratios of specific subclusters increase while the ratios of non-specific subclusters decrease, as shown in A. For each macrophage subcluster, processing reduces the non-specific marker signal while the specific ones are enriched (B). The circle size indicates the positive marker percentage in a particular phenotype, and the circle color indicates the relative change of the positive rate for a particular marker after IMClean pre-processing. **C.** Representative zoomed-in mouse HCC liver tissue section highlighting macrophages colored by cell subpopulation (see color key legend). Subpopulations commonly identified via unsupervised clustering within macrophage compartments in both the raw and processed datasets are M1 macrophages (CD45^+^ F480^+^ CD68^+^), M2 macrophages (CD206^+^ CD68^+^ F480^+^), Proliferating PDL1^+^ macrophages (CD45^+^ PDL1^+^ MHCII^+^ CD68^+^ F480^+^) and CD86^+^ M1 (CD45^+^ MHCII^+^ CD68^+^ F480^+^) macrophages, S100A9^+^ M1 (CD45^+^ CD68^+^ F480^+^) macrophages, and S100A9 (CD206^+^ F480^+^) M2 macrophages. **D-E.** IMClean increases the specificity (D) and sensitivity (E) of dendritic cell subcluster phenotyping; the ratios of specific subclusters with positive scaled expression for a certain marker increase (D). Comparison of marker expressions between raw and IMClean-processed for dendritic cell subclusters showed that IMClean enhances the specific marker signal within cell types. The circle size indicates the positive marker percentage in a particular phenotype, and the circle color indicates the relative change of the positive rate for a particular marker after pre-processing. **F.** Representative case showing spatial distribution of dendritic cell subclusters commonly identified in both datasets in mouse HCC tissue section including Dendritic cells (CD11c^+^) and PDL1^+^ (CD45^+^ CD86^+^ MHCII^+^ CD11c^+^) Dendritic cells (see color key legend).

For macrophage subclusters, we observed that the ratio of cells expressing MHCII (Major Histocompatibility Complex class II), CD86, PDL1, and Ki67 was increased in the biologically specific macrophage subclusters (Fig. 8A). Accordingly, we observed that subcluster specific markers are increased while non-specific markers decreased after IMClean pre-processing (Fig. 8B).

For dendritic cell subclusters, we found that the ratio of cells expressing functional markers such as S100A9, MHCII, CD86, PDL1, S1004A, and Ki67 was increased in the specific PDL1 + dendritic cell subcluster, but not in the more generic ‘dendritic cell’ subcluster (Fig. 8C). Additionally, the ‘dendritic cell’ subcluster showed a high percentage of cells expressing CD11c after IMClean pre-processing (Fig. 8D, circle size) and we observed a positive change in the expression of subcluster specific markers when comparing raw vs. pre-processed data (Fig. 8D, color scale). Visualization specific macrophages and dendritic cells subclusters are shown in Figs. 8E and 8F. This analysis confirms that IMmuneCite can identify different cell types as well as distinguish activation states of different cell types in tumor microenvironments, which usually exhibit a wide diversity. Thus, the identification of rare cell types associated with a certain stage of disease can lead to the identification of biomarkers of response to treatment or predictors of clinical outcomes.

## Discussion

IMmuneCite is an open-source and customizable framework developed for thorough immune focused analysis of spatial proteomic datasets. It enables pre-processing of raw images by using IMClean, which improves the quality of images used to generate a single-cell proteomic dataset by correcting for image artifacts caused by channel spillover, noise, and antibody aggregates (Fig. 1B). Spatial proteomics data are commonly analyzed using unsupervised algorithms, which can be affected by the presence of these artifacts. Thus, the production of optimized, high-quality images is imperative to generate a biologically relevant single-cell dataset to conduct downstream analysis and thus enable meaningful analyses of inflammatory pathologies. Our results demonstrate that IMClean image pre-processing enhances the specificity and sensitivity of immune markers in both metaclusters and subclusters in both murine and human tissue samples, removing clusters reflecting tissue artifacts and non-biological cellular phenotypes, confirming that the IMmuneCite workflow improves data accuracy. IMmuneCite offers a versatile, user-friendly, and reliable computational tool for spatial proteomics data that is adaptable to any antibody panel and capable of capturing multiple complex immune and non-immune cellular phenotypes associated with different diseases. Additionally, the level of phenotype discrimination offered by IMmuneCite allows for the identification of cell populations which can be key features of a certain disease or disease state, as demonstrated by the few discrete PD1 + T-cells which are predictor of TCMR in clinical LT and bolster the concept that the PD1 pathway plays a major role in alloimmunity^52,55–57^. The application of our computational approach to the study of tumor immune microenvironments might enable the identification of cell types associated with therapy response, disease progression and, more generally, patient outcomes, bearing important clinical implication on establishing the level of care.

In this study, IMmuneCite provided an in-depth representation of the intricacy of the alloimmune and tumor microenvironment in both liver allograft rejection and cancer. We showed that IMClean, our Python-based image pre-processing tool, can ameliorate the quality of multiplexed images by correcting for technical artifacts present in IMC images. Indeed, depending on antigen-antibody interactions, spatial proteomics has similar considerations to IHC in order to avoid image artifacts, which include antibody concentration optimization, clonality (monoclonal versus polyclonal), epitope affinity, as well as tissue preservation technique, length and type of fixation, dehydration of the tissue after fixation, and ischemia period (time between tissue collection and fixation)^19–21^. Although IMC is not affected by autofluorescence and background signal, which are typical of fluorophore based technologies, a certain amount of signal spillover or channel crosstalk is still present and can affect experimental results and lead to false conclusions^22^. Channel crosstalk is mainly due to metal isotopic impurity or oxide formation, and can only be partially addressed by a careful design of the antibody panel and selection of highly pure metal isotopes used for antibody conjugation^22–24^. The first step of IMClean allows for channel crosstalk compensation post-acquisition by applying subtraction of the contaminating signal from any channel affected by signal spillover. Chevrier et al. developed CATALYST, an R package that creates a spillover matrix based on signals detected in adjacent channels from separated heavy-metal conjugated antibody placed on a glass agarose slide which is then used during the analysis to correct for spillover^23^. While this might represent a faster and automated way to address the channel crosstalk issue, it has limitations in that spillover compensation of high signal intensities requires the ablation of an antibody matrix every time the marker panel is modified, which comes with additional costs related to both total amount of antibodies required and ablation.

Compared to MAUI, which requires the use of a licensed platform and additional steps to convert mcd into tiff files that can be time consuming, IMClean combines all pre-processing steps in one single Python script, which streamlines the user workload and need for expertise in working with multiple platforms^32^. Compared to IMC-Denoise and SPEX, which focus on noise and aggregate removal and channel crosstalk and denoising respectively, IMClean accounts for all sources of image artifacts and gives the user freedom to decide what correction step to apply. Additionally, while the application of the DIMR algorithm avoids a user-defined intensity threshold or range to identify hot pixels, it cannot remove hot pixels that appear in large clusters. While DeepSNiF remains less accurate compared to supervised denoising methods and requires high bioinformatic expertise, IMClean has been shown to be effective at removing any type of image artifacts with minimal bioinformatics knowledge expertise^28^. By allowing the user to work through each step with minimal code interaction, the focus remains on pre-processing accuracy. IMClean enhances downstream analysis for cell identification and increases the specificity of each marker for the biologically appropriate cell type in data obtained from two different species (Figs. 2 and 6). Additionally, image pre-processing effectively reduces the co-expression of markers from different cell lineages on the same cell, resulting in biologically misleading marker expression patterns and false annotation (Fig. 2F, **Supplementary Fig. S4D** and **S5**). While we have applied IMClean to two IMC datasets, it can be applied to other spatial proteomics technologies such as MIBI and PhenoCycler, which can also be affected by similar image artifacts.

Cell phenotyping in proteomics data is usually performed using manual gating strategies combined with a *priori* knowledge of cell markers or unsupervised algorithms which can be particularly cumbersome in the case of antibody panels with several immune markers with overlapping distribution on multiple cell types. One can often visualize low levels of non-biologic marker expression in clusters annotated based on unsupervised clustering, which may not be evident to readers or reviewers who are not as familiar with the technical details of cluster annotation and solely rely on analysis of assigned cellular identities, which may not be optimized for accuracy^14,58–60^. In this regard, the IMmuneCite clustering algorithm can help in the phenotyping process by performing cell identification in two steps. First, by using a supervised algorithm based on lineage marker expression, the overarching lineage or “compartment” of immune and non-immune cells can be identified (Fig. 1D and 5). Secondly, each cell compartment can be further investigated and dissected to provide details on cell status and function. This step relies on an unsupervised algorithm in combination with functional markers, thus allowing the user to further explore the cell microenvironment and obtain meticulous information about rare cell populations and the overall pathological immune landscape. Detection of rare cell populations, for example PD1 + cells, is particularly relevant to guide immune checkpoint treatment in patients with cancer or discover new biomarkers^13,61–63^. As noted, our clustering approach can be easily modified to accommodate any antibody panel used to stain a wide variety of tissues in different pathological conditions thus accounting for all the various cell populations identifiable.

A previously implemented clustering tool for IMC data was included in Cytomapper, an R/Bioconductor package that allows, among other features, cell labeling based on a hierarchical gating strategy on marker expression values^46^. As such, it is subjective to inter-operator variability ^46^. More recently, Astir (ASsignmenT of sIngle-cell pRoteomics) has been developed *imprimis* for IMC data and relies on a scalable marker-based probabilistic model to assign cell phenotype to proteomics data. Astir uses both, measures of protein expression and a prespecified set of markers, to assign cell phenotype employing a machine learning model^42^. However, Astir has a limited capability in identifying novel cell subsets as it relies on user inputs^42^. Lastly, SIMPLI (Single-cell Identification from MultiPlexed Images) is a tool that allows users to classify cells by choosing between an unsupervised clustering algorithm and a user-defined thresholding method of markers of interest for cell phenotype assignment^43^. Although SIMPLI represents a comprehensive tool for analysis of spatial proteomics data, it does not provide a framework to perform image pre-processing, thus requiring the investigator to have advanced bioinformatics support to work with raw data across various analysis platforms.

The image pre-processing pipeline and the clustering algorithm included in IMmuneCite have been implemented in two different platforms, Python and R, as standalone tools. While this might represent a limitation given that using the entire IMmuneCite framework requires the user to move from one platform to another, it also gives the freedom to perform either image correction or the clustering step and then move into downstream analysis. Another major strength is that both tools are available on free platforms and no license is required. We have provided a step-by-step manual that guides users through the IMClean pipeline. While user-defined parameters are still required for image artifact correction and optimization, our future step is to provide a more algorithmically optimized and automated parameter selection, which would also allow for their application to a set of multiple images and optimization based on tissue type. At this stage, IMmuneCite does not incorporate cell segmentation into the Python pipeline, but it will be incorporated once the Python script for Mesmer becomes publicly available.

In conclusion, the IMmuneCite workflow simplifies an intense data workflow to enable an appropriate quantitative analysis of IMC data, particularly within immune rich disease states. It improves the usability of spatial proteomic data and facilitates cell phenotype identification while reducing incorrect cell phenotype assignment thus ensuring a proper analysis of complex and poorly characterized tissue immune microenvironment.

## Methods

This study was approved by the Health Science Campus Institutional Review board of the University of Southern California (HS-18–00708). Given the retrospective nature of this study informed consent was waived.

### IMmuneCite Framework Description

IMmuneCite is a three-step framework consisting of image pre-processing (IMClean), cell segmentation, and cell phenotyping (https://github.com/julietusc/IMmuneCite_Pipeline). Each of the three steps have been implemented as a standalone tool running on Python, docker, and R, respectively – three freely available platforms. The IMmuneCite framework allows for the pre-processing of raw images and subsequent cell phenotype identification through the integration of previously established and newly developed tools.

### IMClean Pipeline Description

The IMClean pipeline is the first step in the IMmuneCite framework and consists of tiff extraction, signal pre-processing, and stack creation, all of which are executed using Python (v3.8 or greater) in the command line. These processes can be executed either individually through their respective files or altogether by running the master file (a user guide with examples of implemented parameters is available at https://github.com/julietusc/IMmuneCite_Pipeline). User input is required during the signal pre-processing step. The pipeline assumes that standard Python libraries such as os, *pathlib, numpy*, pandas, and *matplotlib* have already been installed by the user. Other packages required will be listed, as appropriate, in each process description below.

#### Tiff extraction.

Single-channel .tiff files are extracted from raw IMC data (.mcd) files using the *imctools* and *tifffile* packages^64,65^. Additionally, the user will also be required to install the *shutil* package^66^.

#### Signal pre-processing.

Pre-processing is done on one antibody channel at a time and involves three steps: background removal (also known as spillover correction), noise removal, and aggregate removal. The first and last steps – background and aggregate removal – are based on the approach used in the MAUI software package developed by the Angelo lab^32^. Additional Python packages required in this step include imctools, *tifffile*, scipy, and *skimage* (also known as *scikit-image*)^64,65,67,68^.

Channel spillover occurs when one channel (“source”) contaminates another channel (“target”), and can either be caused by ionic contamination or by isotopic impurities in the metal stocks that are used for conjugation^32^. In this background removal step, the source channel is pre-processed first by capping, smoothing, and binarizing the signal to generate a mask, all of which require the user to determine parameters. The cap threshold homogenizes the signal intensity of the source channel by accounting for overly bright signals. The threshold, set by the user, determines the maximum value of the source signal, and sets pixel with signals above this threshold to the cap value. A higher cap threshold decreases the number of pixels that are considered contaminating signal. Next, the user selects a Gaussian radius parameter in the radial Gaussian filter, which will blur and smooth the source signal (the higher the radius, the higher the blurring). Finally, the user determines a ‘threshold’ parameter to binarize the signal; any value above the threshold is set to 1 and any value below is set to 0. A higher ‘threshold’ parameter decreases the number of pixels that are considered contaminating signal. This binarized mask of the source channel is then used to clean the target channel; any pixel with a value of “1” will be considered contaminating signal and used to correct for spillover in the target channel. To do so, the user selects a removal value. This value is then subtracted from all pixels in the target channel that have a positive value, “1”, in the source binarized mask. The user then compares the signal of the target channel before and after background removal, with the option of either re-doing this step, selecting an additional ‘source’ channel, or finalizing this step.

Image noise can occur due to a variety of reasons such as instrumentation used during IMC staining, tissue quality, and nonspecific antibody binding, all of which can result in the generation of weak, non-biological signal^22–24^. The noise removal step implements a minimum filter to cap the signal followed by a uniform filter to smooth it. Both of these functions are part of the *scipy.ndimage* package and require the user to select parameters, with the option of setting one or both to zero – meaning the filter is not applied – if desired^67,69^. In certain antibodies, noise tends to have a lower expression than true signal. With the minimum filter, any pixel with a signal level below the designated minimum threshold will be considered noise and set to zero. Signal from noise also has a different origin and thus different characteristics than true signal, with no actual pattern to it. With true signal, neighboring pixels tend to be correlated forming a pattern to represent cell nuclei, membranes, or vessels. By smoothing the signal with the uniform filter, individual signals are replaced with an average signal value of its neighboring pixels’ expression/signal. Any signal from pixels that are not part of a larger pattern (e.g. cellular structures) represent noise and will be set to zero. With the uniform filter threshold, the user determines how many neighboring pixels to consider when calculating the average signal value (a higher threshold corresponds to a larger radius). Once both these parameters have been selected, the before and after noise removal signal is visualized to allow the user to compare noise removal quality, with the option of changing the parameters if needed.

Aggregates can occur due to conglomerations of antibodies that result in high counts in concentrated areas^32^. This can impact downstream analysis as they may lead to false positives in antibody staining. Detection and removal of antibodies requires caution as it could result in removal of true signal; expert knowledge of the tissue, cellular shapes, and how antibodies are supposed to stain is advisable. In this step, users select a Gaussian radius to blur and smooth the image. Image blurring aids in distinguishing true signal from false positives: nearby patches of signal merge together to become a larger structure, such as a cell or vessel, while antibody aggregates tend to remain on their own and will therefore have a smaller size/radius. After blurring, the image gets binarized to create a binary mask and the size of all connected, positive objects is identified. Objects below the size of the ‘size’ threshold, which the user determines, will be considered aggregates and their counts will be set to zero. Here, too, before and after aggregate removal signals are visualized and the user has the option of changing the parameters if needed. Once finalized, the user gets the option of applying these pre-processing parameter settings to all other images.

#### Stack creation.

After signal pre-processing is completed, single-channel TIFF files are exported and saved in the user’s main folder. In this last step, single-channel TIFF files are combined to create a stack of .tiff files per image, as required for cell segmentation in Steinbock. This is again done by using the *tifffile* package^65^.

Once image pre-processing has been completed, the user can continue with the second step of the IMmuneCite framework: **cell segmentation**. Cell segmentation is currently a standalone process implemented in docker using the customizable steinbock toolkit, as developed by the Bodenmiller group.^48^ The steinbock toolkit offers the user the choice between two cell segmentation approaches: Ilastik/Cellprofiler, a supervised pixel classification method, or Mesmer, a completely automated and pre-trained deep-learning-enabled segmentation algorithm^47–50^. The steinbock toolkit also enables the measurement of region properties, aggregated marker intensities, and spatial neighbors.^48^ Cell segmentation outputs include single-cell data – consisting of an expression matrix and morphological and spatial features –, cell segmentation masks, and antibody signal images. These outputs can then be used for cell phenotyping and downstream analysis.

### IMmuneCite’s Clustering Pipeline Description

The IMmuneCite clustering pipeline is implemented as a two-step process – identification of metaclusters and identification of subclusters – and involves a semi-supervised approach implemented in R. Data is first arcsine transformed and then standardized by channel to account for differences in signal intensities.

#### Metaclusters.

Identification of metaclusters involves the use of logical operators and thresholds on the scaled marker expression. First, the user needs to define the lineage markers to be used and all other markers to be considered during clustering and downstream analysis. For each cell, markers are ranked based on their expression and the 3 highest expressing markers are determined. The user can expand the ranking selection to more than 3, if desired. The lineage marker is also identified for each cell by determining which lineage marker has the highest scaled expression. The user then decides which cell populations should be identified as metaclusters – macrophages, CD4+ T-cells, CD8+ T-cells, endothelial cells, etc. – as well as a set of rules on how these metaclusters should be identified. Each rule needs to state the lineage marker used to identify a particular metacluster, whether certain markers need to be expressed above a certain threshold, and whether the 3 highest expressing markers should include or exclude a set of markers. For example, a user may wish to identify the B cell metacluster in human samples as follows: CD20 as the lineage marker, a positive expression of CD20 (that is, the scaled expression of CD20 should be above 0), and the 3 highest expressing markers should exclude markers such as CD4, CD8, CD68, CD163, CD66b, CD31, CK7, and Granzyme B. The latter is to ensure that we exclude cells with a non-biological phenotype (for example, cells that express both B cell and macrophage markers). The user may also use multiple set of rules to identify a particular metacluster. For example, to identify macrophages in human samples, a user may define a set of rules using CD68 as the lineage marker and another set of rules using CD163 as the lineage marker.

We recommend the user not exclude any cells at this stage yet. Rather, cells that may present with a mixed phenotype can be classified as “other” and examined further (heatmaps, staining on tissues, etc.). Metacluster labels should be verified by reviewing concurrent metacluster label and channel expression on tissue sections, and rules and thresholds should be adjusted as needed.

#### Subclusters.

After identifying all desired metaclusters, the user can move on to subclustering. This is done on each metacluster individually and can therefore be performed on all metaclusters or a subset. For a particular metacluster, a subset of the data is extracted to include that metacluster only and used for subclustering. The user then has the option of performing unsupervised clustering right away or subsetting the data even further. For example, for macrophages in human samples, the user may wish to divide all macrophages into M1 and M2 macrophages first using the expression of a particular marker such as CD163 and then perform unsupervised clustering on M1 and M2 macrophages separately. For unsupervised clustering, the user first defines which functional markers should be used. For example, for M2 macrophages in human samples, a user may wish to look at CD16, CD11b, Ki67, and HLADR as well as CD68 and CD163. After defining the markers, unsupervised clustering is performed using the FlowSOM algorithm from the CATALYST package^70^. Although default parameters are set to obtain 9 clusters, the user has the option of modifying these parameters to increase or decrease the number of desired clusters. Clusters are then visualized using a heatmap and can be merged and labelled based on their phenotypic expression.

After performing subclustering on each desired metacluster, subcluster information is incorporated into the main object. This step requires the user to manually identify which subclusters to be incorporated and what their final subcluster label should be. Here, too, users have the option to exclude cells with a non-biological phenotype. After incorporating all individual information into the main object, we advise the user to visualize the phenotypic expression of all subclusters, both combined and by metacluster, to verify subclusters have been labelled and combined appropriately. Subclusters can also be verified by reviewing concurrent subcluster label and channel expression on tissue sections.

### Sample Description

Human liver samples analyzed in the present study have been identified using our institutional database as described previously^71^.

#### IMC staining and ablation.

Formalin-fixed paraffin embedded (FFPE) tissue sections of liver biopsy specimens were stained using a customized 22-marker panel and ablated with techniques described previously^6^.

#### Validation Data.

A publicly available dataset containing 12 liver samples obtained from mouse HCC models and stained with a 35-antibody IMC panel was used to validate the IMmuneCite framework^14^. Images were first pre-processed using the IMClean pipeline and then segmented using the same approach as outlined above, with CD68 used as the ‘source’ channel during channel spillover correction. Raw data was also segmented. Raw and pre-processed data were then both loaded into R for single-cell phenotyping using the IMmuneCite clustering algorithm.

### Image pre-processing using the IMClean pipeline.

Pre-processing was implemented in three batches by clinical outcome (NR, TCMR, CR) to account for staining differences between disease states. CD68 was used as the ‘source’ channel for spillover correction; noise removal and channel aggregate removal steps were implemented individually on each channel. After pre-processing, cell segmentation was performed on both the ‘raw’ and the pre-processed datasets using Mesmer (DeepCell) and following the Bodenmiller Steinbock pipeline^48^.

### Phenotypic clustering using the IMmuneCite clustering pipeline.

#### Humans:

Cell segmentation outputs were loaded separately into R to perform phenotypic clustering and downstream analysis, first on the raw dataset and then on the pre-processed one. Data were arcsine transformed and standardized by channel to account for differences in signal intensities. Following our IMC pipeline, we used 10 lineage markers (CD4, CD8, CD20, CD68, CD163, CD11b, CD66b, CD31, CK7, and CD138) to identify the following 10 metaclusters: CD4^+^ T-cells, CD8^+^ T-cells, B cells, macrophages, monocytes, plasma cells, neutrophils, endothelial cells, cholangiocytes, and hepatocytes. Labelling accuracy was verified by reviewing concurrent metacluster label and channel expression on tissue sections. Masks were used to visualize cell labels (*cytomapper::plotCells*)^46^. TIFF images were scaled, and channel signals were normalized and visualized individually (*cytomapper::plotPixels*). Subclustering was subsequently performed on the five most relevant immune metaclusters (CD4^+^ T-cells, CD8^+^ T-cells, B cells, macrophages, and monocytes) and the three non-immune metaclusters using a semi-supervised approach. CD8+ T-cell, B cell, monocyte, hepatocyte, endothelial cell, and cholangiocyte subclusters were identified via FlowSOM. For each, the resulting 9 clusters were visualized alongside channel expressions on a heatmap and merged and annotated according to their phenotype. CD4+ T-cells were first divided into CD3 high and CD3 low. FlowSOM clustering was then done on each of the two groups separately. Resulting clusters were merged and annotated to obtain CD4+ T-cell subclusters. The CD4+ Treg subcluster was further divided into HLADR+ CD4+ Tregs and HLADR− CD4+ Tregs. Macrophages were first divided into M1 and M2 macrophages based on their CD163 expression and then clustered separately using FlowSOM to obtain all macrophage subclusters.

#### Mouse:

Metacluster lineage markers include immune markers (CD3, CD4, CD8, B220, CD68, CD11c, F480, CD206, CD11b, Ly6G, MHCII, CD161) and non-immune markers (CD31, CD29, Ecad, aSMA). Functional or phenotypic markers used in subclustering include PD1, FoxP3, Ki67, Granzyme B, PDL1, CD86, S100A9, and S100A4, among others. Identified metaclusters include CD4+ T-cells, CD8+ T-cells, macrophages, myofibroblasts, B cells, dendritic cells, PMN, epithelial cells, endothelial cells, and other non-immune cells. Subclustering was performed on select metaclusters (CD4+ T-cells, CD8+ T-cells, macrophages, B cells, and dendritic cells).

### Statistical Analysis

Raw and pre-processed data were standardized after integration to allow for better comparison. Signal intensities were compared across the two datasets by channel. Dimensionality reduction was performed using t-Distributed Stochastic Neighbor Embedding (t-SNE) to visualize metacluster differences by clinical outcome across both datasets^72^. Heatmaps were used to visualize phenotype expression by meta- and subcluster across both datasets. To determine differences in cell proportion after pre-processing, the relative change of the median cell proportion by patient (meta- and subcluster) was calculated. Boxplots were used to visualize cell proportion differences by subclusters across both datasets. For each dataset, the positive marker percentage in a particular phenotype was determined. The relative change of these percentages was then used to analyze phenotype sensitivity after pre-processing. The ratio of positive marker percentage within a meta/subcluster vs. all other meta/subclusters was calculated for each dataset. The relative change of the ratios was then used to analyze phenotype specificity after pre-processing. The median fold change was used to calculate the difference in median expression by marker between raw and pre-processed data. The proportion of cells with a mixed phenotype was calculated by analyzing the highest expressing markers in each cell; the relative change was then visualized between raw and pre-processed data. Seeds were set to allow for reproducibility. All statistical tests were carried out in R (v 4.2.2).

## Data Availability

All data needed to evaluate the conclusions of the present work are contained in the paper and/or the Supplementary Materials. Data and scripts to reproduce the findings of this study are available on our Github (https://github.com/julietusc/IMmuneCite_Manuscript). Raw data is available upon reasonable request to the corresponding author. The external dataset used as part of validation analysis is available from Zabranski, DJ et al. (https://doi.org/10.1002/hep.32707).
